# Unpacking Contexting and Institutionalizing as Complex Sustaining Practices: Comment on "Sustaining Knowledge Translation Practices: A Critical Interpretive Synthesis"

**DOI:** 10.34172/ijhpm.2023.7523

**Published:** 2023-02-13

**Authors:** Ninna Meier

**Affiliations:** Department of Sociology and Social Work, Aalborg University, Aalborg, Denmark.

**Keywords:** Knowledge Translation, Context, Organizational Change

## Abstract

This article discusses the work of Borst et al in which they suggest ‘sustaining work’ as a term that covers the efforts of actors to sustain the use of health research in policy and practice through three practices. I suggest that two of these, *contexting* and *institutionalizing*, need to be further unpacked to understand how and why they are important for sustaining work in knowledge translation (KT). To contribute here, I discuss KT as processes of organizational change that occurs within and across organizations, often involving actors with different views on and approaches to the use of health research in policy and practice. These actors will likely have very different understandings of what the context for using research is and they are likely be members of competing or conflicting institutions. Future research needs to take such elements into account to improve our understanding and practice of sustaining work.

 Why is the use of health research in policy and practice so difficult to sustain? Borst and colleagues’^1^ article adopts a critical interpretive synthesis approach to provide answers to this important question. Based on a review of health policy and systems research and Science and Technology Studies literature on sustainability, Borst et al aim to identify and explain knowledge translation (KT) processes, activities, and efforts that facilitate the sustaining of KT practices (p. 2). In doing this, they make several contributions: they propose that the literature on sustainability of KT has shifted from sustainability as a relatively stable end goal towards sustain*ing* as the ongoing work actors engage in to make and keep KT practices productive. These efforts, they suggest, can be understood as ‘sustaining work,’ which consists of the interplay between translating, contexting, and institutionalizing (p. 4). Last, Borst et al convincingly argue that while current research point to contexts and institutions as essential factors for sustaining the use of health research in policy and practice, there is a need to move beyond lists of factors and towards a more dynamic and practice-based approach. These contributions underscore the significance of focusing on the mundane, everyday work activities with which actors sustain the use of health research in policy and practice. However, in their approach Borst et al leave at least two central aspects of their question unaddressed: first, they do not discuss how KT can be understood as a process that entails organisational change and in which multiple organisations as well as actors are involved. Given the focus of their critical interpretive synthesis this is to be expected but it inadvertently assigns important organizational aspects of the processes within KT and sustaining work to the background.

 Second, Borst et al do not fully discuss what ‘contexting’ and ‘institutionalizing’ might involve in practice or how actors contextualize and institutionalize differently, and perhaps conflictingly, depending on their professional background and socialization, their position and role within their organization, their prior experiences, and their approach to the current process more broadly. While central to KT practices, the terms ‘contexting’ and ‘institutionalizing’ need to be further examined to fully understand how and why they are important for sustaining work.

 In my view, the two aspects addressed above are connected in ways that are important for how we understand and can research how people engage in sustaining work.

## Knowledge Translation as Organizational Change

 KT from research to policy and practice can be understood as a process of organisational change that occurs within and/or across organizations, depending on how you approach the change-action-context relationship.^2^ In practice, the process of moving from knowledge to action involve actors with different views on and approaches to the use of health research in policy and practice. These actors will likely have very different understandings of what ‘the context’ for using research is and should be. They are also likely to be members of competing or conflicting institutions where sources of legitimacy vary.

 Research into organizational change has mostly focused on change as strategic, planned, and managed but increasingly perspectives that acknowledge the complex and emergent nature of change processes gain recognition for being able to offer explanations of why and how unintended consequences can arise, eg, during a process of implementation of health technology.^3,4^ Here, as with KT, understanding context is crucial.^5^ The term *context* means weaving or knitting together, to make a connection between a phenomenon and that which we deem relevant for understanding it.^6^ In this understanding, context is what helps us make sense of a change process as its frame or background, eg, in relation to the processes involved in sustaining the use of health research in policy and practice. Drawing on the Science and Technology Studies literature, Borst et al acknowledge the ongoing analytical construction of context (p. 7) but focus mainly on groups of actors in networks and how they construct contexts that work. Curiously, they do not address the obvious potential for disagreements, misunderstandings, and conflicts in such processes, even though this aspect of contexting offers an explanation that might account for why it is so difficult to sustain KT. When many actors from different professions and organisations need to be involved and carry out sustaining work, there is no one single context that we can assume all involved agree on and relate their sustaining efforts to through contexting. Rather, every actor or position in a network comes with a vantage point from which they view and engage with the process even if they share an overall goal of improving patient care. This vantage point can be influenced by factors such as professional background, expectations, experience, and role in the KT process. Because context is not fixed, lack of alignment in sustaining efforts can arise along several lines within or across healthcare organizations and require attention throughout the process. Barriers such as insufficient time, lack of motivation, or aspects of the organizational culture are also likely to influence the process.

 Sensemaking research is occupied with understanding how actors individually and collectively enact and make sense, for instance of the organisational change processes they are part of.^7^ This research points to the role of emotions in such processes and propose that actors’ emotions (eg, fear, joy, disappointment or excitement) are important for understanding how they are part of a change process.^8^ Emotions are also essential to understanding actors’ engagement with institutions through institutional work.^9^ Borst et al write briefly about institutional work and refer to their third process as institutionalizing. Within institutional work theory, the concept of institutional maintenance refers to work people do in various ways to maintain institutions, for instance through practices of policing, enabling, or deterring others^10^ but people can also do institutional work to create, change, or disrupt institutions, possibly practiced alongside their efforts to maintain their ‘own’ institution. In this view, we can understand sustaining the use of health research in policy and practice as a form of institutional maintenance in one context (eg, healthcare) and as institutional change in other contexts (eg, social work). This is because the purpose and practical consequence of institutionalizing are not fixed either, they must be understood relative to what is constructed as context (which among other things depends on one’s position and approach). The view that Borst et al adopt is one where institutionalizing involves ‘actively and strategically using institutions to sustain KT practices’ (p. 8) and they propose that the sustaining of KT depends partly on the extent to which actors ‘use institutions to make and keep their KT practices productive’ (p. 8). As with the processes of contexting, this approach seems to assume that actors agree on which institutions to use and how, or at least that their institutionalizing is coordinated and directed towards a shared goal of keeping KT practices productive. In short, an essential question seems to center around how actors coordinate and align their KT efforts and practices to become *collective* efforts that are sustained through eg, organisational routines and structures.

 I hope to have shown that the contexting and institutionalizing aspects of sustaining work contain much more complexity than is evident on the surface. Acknowledging the complexity of the practices that these labels refer to can potentially further theorization and empirical research into the use of health research in policy and practice, and hopefully also benefit practitioners. Paradoxically, whenever we produce research about context-dependent and complex practices that can be interpreted and enacted differently by people, we risk producing yet another ‘knowledge to action gap.’ In this case, actors tasked with sustaining the use of health research will – after reading Borst et al – know that contexting and institutionalizing are essential for sustaining work, but they are left to figure out precisely how to *do* this sustaining work in practice. Unpacking the complexity of terms such as contexting and institutionalizing will not solve this challenge, but we should strive towards addressing the gap in hopefully useful ways. For instance, future empirical research into the practices with which actors continually enact contexts for KT sustainment can specify *how* the research focus on contexting or institutionalizing as practices. One way could be to analyze patterns in how contexts are made and enacted by actors across different positions in a network or investigate the reciprocal aspect of contexting when people practice sustaining work across boundaries. Are contexting or institutionalizing practices of formal managers, for instance, more successful and accepted by others than those of people in less powerful roles? Or does this depend on the institutional setting, ie, do the legitimacy of clinical opinion leaders influence their ability to enact a shared context for KT that sustains use, as the work of Dopson and Fitzgerald^11^ would suggest? A different avenue could focus on the role of emotion and particularly how emotion regulation relates to sustaining work, thus connecting to the literature of institutional work. Future research could also explore contexting or institutionalizing as ongoing collective processes, for instance focusing on how sustaining is embedded in existing infrastructures of knowledge and expertise. All these questions are avenues for future research that the work of Borst el al has opened and that KT research can benefit from pursuing.

## Ethical issues

 Not applicable.

## Competing interests

 Author declares that she has no competing interests.

## Author’s contribution

 NM is the single author of the paper.
